# Localisation of epileptic foci using novel imaging modalities

**DOI:** 10.1097/WCO.0b013e328363372c

**Published:** 2013-07-03

**Authors:** Alessio De Ciantis, Louis Lemieux

**Affiliations:** Department of Clinical and Experimental Epilepsy, UCL Institute of Neurology, Queen Square, London, UK

**Keywords:** electrophysiology, epilepsy, ictiogenesis, imaging, localization, seizures

## Abstract

**Purpose of review:**

This review examines recent reports on the use of advanced techniques to map the regions and networks involved during focal epileptic seizure generation in humans.

**Recent findings:**

A number of imaging techniques are capable of providing new localizing information on the ictal processes and epileptogenic zone. Evaluating the clinical utility of these findings has been mainly performed through post-hoc comparison with the findings of invasive EEG and ictal single-photon emission computed tomography, using postsurgical seizure reduction as the main outcome measure. Added value has been demonstrated in MRI-negative cases. Improved understanding of the human ictiogenic processes and the focus vs. network hypothesis is likely to result from the application of multimodal techniques that combine electrophysiological, semiological, and whole-brain coverage of brain activity changes.

**Summary:**

On the basis of recent research in the field of neuroimaging, several novel imaging modalities have been improved and developed to provide information about the localization of epileptic foci.

## INTRODUCTION

Seizures are the defining feature of epilepsy. They not only induce paroxysmal disability when they occur, but are also thought to be responsible for long-lasting functional impairments. Understanding how seizures arise (ictiogenesis) and their effects on normal brain function in humans is the key to curing epilepsy. For the affected individuals, uncontrolled recurring seizures and the adverse effects of antiepileptic drugs (AEDs) are the key quality-of-life determinants and have an important developmental impact in affected young people. Therefore, we need earlier and better therapeutic interventions to prevent or stop seizures, and to reduce the need for medication. The fact that seizures persist in a large proportion of patients who undergo surgery can be partly explained by our inability to characterize the ictal onset using current electrophysiological and imaging techniques but also by our lack of understanding of the nature of the ictal onset zone or network. Furthermore, surgical success does not prove that the epileptogenic zone or network has been properly characterized; it may be that network disruption prevents seizure generation. Crude disruption surgery (callosotomy) has had limited benefit [1,2]. Chronic deep brain stimulation may reduce seizure and interictal spike frequency in some cases [3], with greater efficacy in cases with electrodes placed within ictiogenic areas [4,5]. The future success of stimulation, local drug injection [6,7], or disconnections [8] rests on improved characterization of the area or network of seizure onset. There is, therefore, a pressing need to improve noninvasive means of identifying brain areas that are involved in seizure initiation, propagation, and cessation. This is important for individual patients who may be considered for curative surgery, but also because in order to understand how seizures begin, we must know where they begin.

**Box 1:**
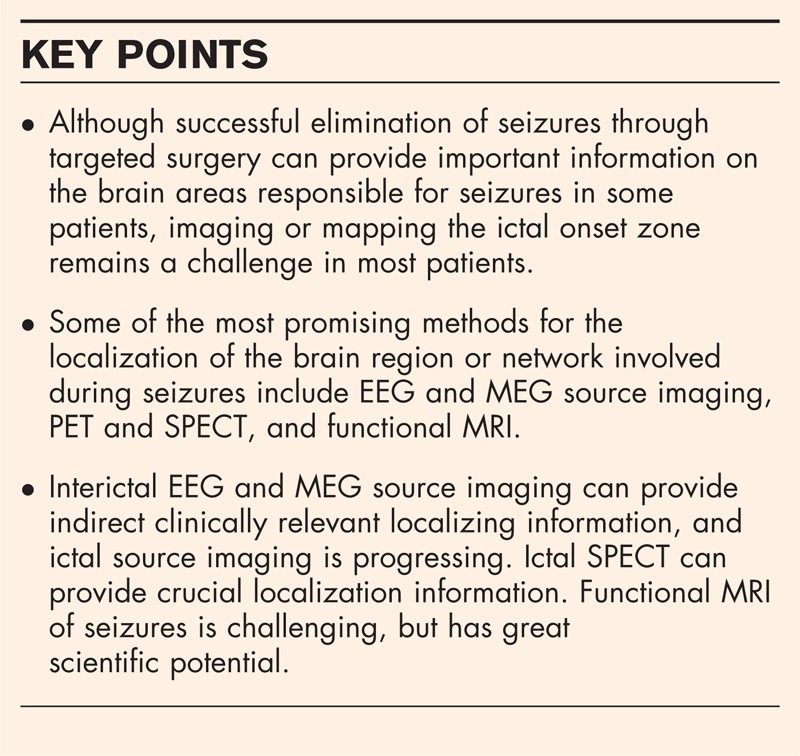
no caption available

The temporal aspect of ictal imaging is particularly interesting because the dynamics of all seizures are at the very least composed of three elements: transition from the interictal state (onset), ictal phase (seizure itself), and transition out of the ictal phase (cessation); the ictal phase itself usually lasts many seconds, and is often characterized by semiological and/or electrophysiological changes. The question of the focal or network nature of ictiogenesis [9] is also relevant because of the advent of techniques, such as functional MRI (fMRI), capable of imaging the whole brain, in contrast to more localization hypothesis-driven techniques such as intracranial electroencephalogram (EEG), usually associated with very limited coverage, and EEG and magnetoencephalography (MEG) with their specific spatial sensitivity biases (neocortical sources). The question of the optimal or ideal marker of ictal events and ictiogenic generators being open, practically any technical development that can provide information *in vivo* in the form of some spatially encoded signal at the time scale of seconds must be considered a potential source of useful information. Therefore, a considerable array of techniques derived from a variety of physical and chemical effects have been applied to the problem, from electrical potentials (EEG, source imaging), nuclear magnetic resonance (fMRI), and radioisotope-tagged markers (PET and single-photon emission computed tomography, SPECT). Certain aspects of most epileptic seizures, such as movement, pose practical challenges for imaging. Beyond the fundamental issue of sensitivity is that of specificity of the localizing information obtained, the evaluation of which remains a challenge due to the dubious quality of the so-called gold standard of localization in many patients and fundamental study design issues [10^▪^].

## SCOPE OF THIS REVIEW

This review focuses on the latest advances in neuroimaging specifically relevant to localization of the brain region or network involved during seizures. In other words, using the terminology proposed by Luders *et al*. [11] for brevity, we will concentrate the discussion on investigations that set out to define the seizure onset zone (SOZ), preferably through the direct observation of seizures or of specific pre or postictal periods. Conversely, studies designed to localize the generator(s) of interictal epileptiform discharges (IEDs), the so-called irritative zone, as a putative surrogate of the SOZ will be mentioned when most relevant (for example, when validated against good independent SOZ localization information), as will studies focused on the structural or physiological substrate or sequelae of seizures. Emphasis is put on studies published in 2012–2013. For an overview of the role of imaging in the management of patients with drug-resistant epilepsy, see study by Duncan [12].

## EEG AND MAGNETOENCEPHALOGRAPHY SOURCE IMAGING

The EEG reflects the synchronous activity of pyramidal neurons over neocortical areas of the order of 6–10 cm^2^[13]. Because of this relatively direct link with neuronal activity and excellent temporal resolution, EEG particularly when recorded within the brain is considered the gold standard at least in terms of sensitivity to epileptic activity in the vicinity of the electrodes. However, recent microarray measurements in humans have revealed abnormal peri-ictal electrophysiological neural firing patterns and preictal changes in the heterogeneity of neuronal spiking patterns beyond the ictal onset zone [14]. Furthermore, blood flow changes have been observed that may precede changes on intracranial EEG (see ‘fMRI’ section).

Although in 1853 Helmholtz proved that the inverse problem of EEG is fundamentally ill-posed (lacks a unique solution) [15], numerical and mathematical techniques have been employed to estimate and localize the generators of epileptic EEG (and MEG) discharges since the 1950s, relying on simplifying assumptions or constraints to render the problem solvable [16]. A number of important methodological developments have taken place over the last decade, including the crucial realization of the importance of adequate electrode coverage and more sophisticated distributed source models [16,17]; a pragmatic way of tackling the multiplicity of source models available is to compare the solutions, which may help assess confidence, with the risk of making interpretation more complex [18^▪^]. EEG source imaging (ESI) and magnetic source imaging (MSI) of IEDs have been validated against invasive recordings, for example [19], shown to impact on the planning of invasive EEG [20], and become an integral part of the presurgical clinical protocol in some centers [21]. MSI can also provide clues toward localizing the irritative zone especially in epileptic patients with negative MRI. Schneider *et al.*[22^▪▪^] published the first large study to investigate the utility of MSI specifically for localizing the epileptogenic zone and predicting epilepsy surgery outcome in nonlesional neocortical focal epilepsy. Recently, MSI was used specifically for localizing the epileptogenic zone and predicting epilepsy surgery outcome in nonlesional neocortical focal epilepsy. A 30–60 min resting state recording using a 306-channel system was performed and a minimum of five spikes was required for a localization result. The MSI finding was compared with the intracranial EEG (ICEEG) findings. The authors demonstrated both that MEG is a useful tool to localize the epileptogenic zone and determine the site of surgical resection in MRI-negative patients and also that a sublobar concordance of ICEEG and MSI was significantly correlated with a favorable surgical outcome. Wu *et al.*[23^▪^] provided further evidence that epilepsy surgery may be an option for pharmacoresistant patients with negative MRI using MEG at rest. The authors set a threshold of 5 or more IEDs and used a single moving dipole model. Among 12 patients (66.7%) with monofocal MEG localizations, 10 (55.6%) presented positive postoperative outcomes. Conversely, all five patients with multifocal MEG localizations achieved bad outcomes.

Ictal source imaging is particularly challenging due to the relative rarity of the events (compared to IED) in most patients combined with limited instrument access time (especially for MEG), the effects of head motion on data quality, and the modeling issues related to possible rapid spread (complex, extensive generators). For ESI, it has been recommended to record from a minimum of 64 recording channels [24]; this can only be practically obtained using EEG caps, which are not part of standard clinical practice. Furthermore, in addition to possible EEG masking by muscle artefacts, the preponderance of fast, low amplitude activity at ictal onset, in contrast to IED, presents the investigator with additional data analysis problems: onset identification and low signal-to-noise. One solution is to perform source estimation continuously throughout (and possibly prior to) the event, such as projecting the EEG onto a number of fixed sources for visual interpretation [25,26] or using more quantitative techniques to tease out the dynamics from the estimated sources [27]. Koessler *et al.*[28] performed arguably the most thorough evaluation of ictal ESI to date in nearly ideal recording conditions (64 channels; duration: 1 h to 2 days) and with invasive EEG validation, in 10 patients. Four forms of early ictal activity at onset were considered (ictal spikes, rhythmic activity, paroxysmal fast activity and artefact-obscured) for ESI performed using equivalent current dipoles, multiple signal classification (MUSIC), and distributed source models LORETA (low resolution electromagnetic tomography) and standardized LORETA (sLORETA); see [17] for a review of source models. The equivalent current dipole solutions had the highest degree of concordance with the results of depth EEG; the authors observed that rhythmic activity tends to originate from propagation areas, confirmed on depth EEG.

In a recent ictal MSI study, Medvedovsky *et al.*[29] managed to record seizures in 20% of patients undergoing MEG over periods of time extending from 1 to 40 h. Using multiple equivalent current dipole to represent source activity for earliest epileptiform signal, the resulting distribution of sources was in good agreement with the distribution of depth EEG channels showing epileptiform activity at the beginning of the seizures, at the sublobar level. The authors rightly note that this assessment is limited by depth EEG's limited coverage, which is itself biased by the fact that the implantations were partly based on the results of IED MSI.

## PET AND SINGLE-PHOTON EMISSION COMPUTED TOMOGRAPHY

SPECT can map ictal blood flow changes by the timely injection of a radiolabeled tracer and statistical comparison of the resulting images and interictal images [30]. The technology is widely available and when performed satisfactorily, ictal SPECT can contribute significantly to the localization of the SOZ, and is particularly useful in MRI-negative cases [31]. The technique produces a single image per injection and, therefore, is ill-suited to the investigation of ictal dynamics.

In a recent study, Schneider *et al.*[32^▪▪^] evaluated the utility of ictal SPECT and interictal MSI, each compared with ICEEG, to localize the epileptogenic zone and predict epilepsy surgery outcome in patients with nonlesional focal epilepsy. Approximately 50 min of resting state MEG activity was recorded in 14 patients. A minimum number of five IEDs were required for a valid MSI localization result. The MEG data were then coregistered to the preoperative MRI to evaluate the anatomic localization. Injection of radiotracer was commenced immediately after either clinical onset or electroencephalographic seizure onset and ictal SPECT was obtained immediately after injection. Interictal SPECT was performed following seizure-free periods of at least 24 h and subtraction ictal SPECT co-registered to MRI (SISCOM) was performed. The authors established that in cases of concordant results, ICEEG-MSI could provide more information for localizing the epileptogenic zone compared to ICEEG-SISCOM and that MSI is better than SISCOM in predicting postsurgical seizure freedom.

PET is much more complex, expensive, and less available than SPECT. Interictal PET using 18F-fluorodeoxyglucose (FDG) is able to reveal abnormally low metabolism in the epileptic focus, although this information is redundant when a lesion is shown on MRI, which is concordant with other electroclinical data [12].

Desai *et al.*[33^▪^] compared interictal PET and ictal subtraction SPECT with subdural and depth electrode recordings in patients with medically intractable epilepsy. The authors demonstrated that both interictal PET and ictal subtraction SPECT studies can provide important information in the preoperative evaluation of these types of patients, but the latter appears to be more sensitive. They also found an increased concordance of both PET and SPECT findings with those from intracranial EEG in MRI-positive patients and those with temporal lobe epilepsy.

## FUNCTIONAL MRI

fMRI of seizures is a more recent development. The technique has attractive features for the study of epileptic activity in humans: it is noninvasive and capable of revealing localized signal changes linked to fluctuations in brain activity at a nominal sampling rate of the order of 1 s, throughout most of the brain, with uniform sensitivity and a spatial resolution of a few mm or greater (depending on the scanner field strength, acquisition sequence, and other instrumental aspects). It is commonly stated that the hemodynamic nature of the blood oxygen level-dependent (BOLD) signal means that fMRI provides less direct observations of neural activity than EEG or MEG. However, the ability to detect brain activity changes other than reflected on EEG (for example, associated with increases in blood flow, but not synchronous excitatory post-synaptic potentials [34]) or semiologically, opens potential new horizons on ictal dynamics. In fact, this complementarity is one of the main motivations for attempting fMRI of epileptic activity, in addition to the more uniform spatial sensitivity than electrophysiological techniques. A number of resting-state fMRI studies of IED in focal epilepsy have demonstrated the technique's potential clinical relevance [35,36,37^▪▪^].

In addition to the time constraints imposed by scanner access, which are similar to PET and MEG in most settings, there are specific challenges for performing fMRI of seizures: first, patient safety due to the particularly limited space (risk of injury) and access. Second, fMRI sensitivity can be severely degraded by head motion and physiological noise (linked to respiration, heartbeat, etc.). These can be addressed partly through careful patient selection, the use of head restraint, and the measurement and inclusion of confound effects into the fMRI analysis; a review of the development of fMRI for the study of epileptic seizures can be found in the study by Chaudhary *et al.*[38^▪^].

Due to the technique's semiquantitative and essentially dynamic nature, fMR images are statistical maps that can be seen as resulting from the voxel-by-voxel comparison of scans acquired in a given state of interest to scans in the ‘baseline’. Therefore, each scan must be labeled according to the participant's brain state at the time of its acquisition. Whereas early ictal fMRI studies relied on visual observation of the patient for this purpose [39–41], the advent of time-locked EEG recording during fMRI, and subsequently video, have facilitated event detection and enriched fMRI scan time series labeling on which predictors of the BOLD changes can be based [42].

By including effects related to interictal (IED; represented as very brief events) and ictal events (e.g., represented as ‘blocks’) into the analysis, one is able to study differences in the generators within a rigorous statistical framework. Using this approach, it was found that BOLD changes in response to epileptiform activity vary with the type of malformation of cortical development. In particular for nodular heterotopia, there was a discrepancy between the structures involved during interictal and ictal discharges [43]. By segmenting seizures into ‘ictal phases’ representing early ictal EEG change, clinical seizure onset, and late ictal EEG change, respectively, we may be able to detect BOLD changes more specifically associated with each [44]. For example, using the same approach (with most patients at rest; in a few, seizure triggers were employed) combined with additional semiological information from synchronized video, and by including an additional preictal phase, Chaudhary *et al.*[45^▪▪^] demonstrated often widespread phase-specific BOLD maps with different degrees of concordance with the SOZ defined through other electroclinical data across the group, most concordant for the ictal onset phase than later phases, suggesting propagation effects. Notably, preictal changes were common, and had a fairly characteristic time signature and tended to be more widespread than at onset. In an attempt to go ‘beyond’ mapping (of BOLD changes correlated with ictal events), there are a few case reports looking into the causal structure of brain networks involved during seizures using fMRI [46,47^▪^].

## CONCLUSION

Although some advances in imaging over the last decade have had a concrete and measurable impact on the identification of surgical targets in selected patient groups, and may have contributed to surgery becoming an option in a greater proportion of cases, the overall outlook for patients with drug-resistant epilepsy has not changed dramatically. Furthermore, we do not know why seizures persist in some patients following surgical resection. Eliminating seizures while preserving normal brain function will require ever more precise identification of the region or regions responsible for seizures and their relationship with eloquent systems. This raises the question of the nature of ictiogenic systems: network or region? Without doubt, the focus model must be accurate in some patients. Concerning the consequences of seizures, is transient and long-term brain function impairment related to spread?

The availability of imaging data (and ever more sophisticated EEG and MEG source imaging techniques) suitable to map seizure dynamics throughout the brain (particularly fMRI, particularly as faster fMRI sequences capable of sub-second whole-brain sampling are being developed [48]), including the interictal–ictal transition, should help shed light on the above questions. Furthermore, the fundamental question ‘Why and how do human seizures occur, and stop, when they do?’ can only be answered if we know where such processes occur. One can, therefore, envisage scientific progress in the form of ever more sensitive and rapid whole-brain imaging, perhaps combined with sophisticated biophysical models of brain networks, allowing us to ‘home in’ on the human ictiogenic process, with important potential positive repercussions for all people affected by recurring seizures.

## Acknowledgements

None.

### Conflicts of interest

There are no conflicts of interest.

## REFERENCES AND RECOMMENDED READING

Papers of particular interest, published within the annual period of review, have been highlighted as:

▪ of special interest
▪▪ of outstanding interest

Additional references related to this topic can also be found in the Current World Literature section in this issue (pp. 449–450).
